# Study of rotavirus genotypes G and P in one Egyptian center-cross-sectional study

**DOI:** 10.1186/s13052-024-01810-x

**Published:** 2024-11-14

**Authors:** AbelRahman Eid Mahmoud, Maysaa El Sayed Zaki, Eman Hamdy Mohamed, Ehab M. Fahmy, Sanaa Samir Mohamed Hamam, Mona Abdellatif Alsayed

**Affiliations:** 1 Pediatrics Department, Faculty of Medicine, Mansoura University, Mansoura, Egypt; 2 Clinical Pathology Department, Faculty of Medicine, Mansoura University, Mansoura, Egypt; 3 Clinical Pathology Department, Faculty of Medicine, Beni Suef University, Beni Suef, Egypt; 4 Medical Microbiology and Immunology, Faculty of Medicine, Helwan University, Helwan, Egypt; 5 Medical Microbiology and Immunology, Faculty of Medicine, Menoufia University, Menoufia, Egypt

**Keywords:** Children, Rotavirus, Genotypes G, Genotypes P, Nested PCR

## Abstract

**Background:**

Rotavirus-associated gastroenteritis is a common health problem in children, different variations of rotavirus genotypes differ according to geographic locations and the practice of wide-scale vaccination. Therefore, the present study aimed to detect both the G and P genotypes of rotavirus in children ≤ 5 years old in one center in Egypt as a cross-sectional study, to correlate the genotypes with various demographic and clinical data in infected children and to evaluate the common mixed genotypes G and P in infected children.

**Method:**

The cross-sectional study included children with acute gastroenteritis ≤ 5 years old from January 2023 till March 2024 recruited from Mansoura University Children’s Hospital, Egypt based upon laboratory diagnosis by exclusion of bacterial and protozoa pathogens. The stool samples were obtained from each child and subjected to detection of rotavirus antigen by enzyme-linked immunosorbent assay (ELISA) followed by genotypes identification of G and P genotypes by nested polymerase chain reaction (PCR).

**Result:**

A nested PCR study for rotavirus genotypes revealed that G1 was the most common genotype (24.7%) followed by G2 (21.1%), G3 (20%), G9 (20%), and G4 (14.1%). The genotyping of the P genotype revealed that P9 was the commonest genotype (24.7%), followed by P4 (21.2%), P10 (20%), P8 (17.6%) and P6 (16.5%). The commonest combined genotypes of G and P were G1P4 (85.7%), G3P8(88.2%), followed by G2P6 (77.8%) and G9P9(76.5%) and G4P9 (66.7%) followed by G4P10 (33.3%), G9P10(23.5%), G2P10(22.2%), G1P10 (14.3%), G3P10(11.8%). The distribution was significant (*P* = 0.001). The positive rotavirus antigen was more frequently detected in females (55.3%) than males (44.7%, Odd ratio 0.2, 95% CI 0.22–0.71, *P* = 0.001). There was a significant association between the summer season and positive rotavirus antigen (*P* = 0.001) and rural residence of the patients (Odd ratio 6,9 95%CI 3,5-13.5, *P* = 0.001). The significant associated clinical sign with positive rotavirus antigen was fever (Odd ratio 3,3, 95%CI 1,8-6.05, *P* = 0.001). The genotypes G and P were significantly associated with positive rotavirus antigen as all cases positive by antigen had been detected by nested PCR with the commonest genotypes G4 (24.7%, *P* = 0.001) and genotype P9 (24.7%, *P* = 0.001).

**Conclusion:**

The present study highlights the common genotypes of rotavirus at one center in Egypt, G1, G2, and G3 were the commonest G genotypes. As regard genotype P the commonest genotypes were P9, P4, and P10. The commonest combined genotypes were G1P4, G3P8, G2P6. There was no effect of the practice of rotavirus vaccination at limited rates at private health sections as the rotavirus is still a major pathogen of acute gastroenteritis in children. There is a need for the inclusion of rotavirus vaccination in the national program of children vaccination in Egypt.

## Introduction

Acute gastroenteritis (AGE) frequently causes gastrointestinal disorders in both industrialized and developing countries. The clinical manifestation of acute gastroenteritis is loose or watery diarrhea, characterized by the occurrence of three or more bowel movements within 24 h Additional symptoms may encompass emesis, pyrexia, febrile signs, or abdominal discomfort [1]. In the Middle East and North Africa countries (MENA) region, diarrheal illnesses cause more than four million episodes and almost 34,000 fatalities annually [2]. Moreover, in children under five, diarrhea is the second most common preventable cause of death [3, 4]. Although AGE can be caused by various bacterial, viral, or parasitic agents, viral infections account for most AGE cases, especially in children [5]. Rotavirus, norovirus, astrovirus, adenovirus, and sapovirus are the most often found viruses linked to AGE. There are other ways that infectious AGE might spread, but the fecal-oral pathway is largely responsible for disease propagation [6].

Rotavirus (RV) is an important pathogen causing acute gastroenteritis in children under five years of age that ranges from mild to severe. RV genome comprises 11 segments of double-stranded RNA, which may generate new variants by reassortants [7, 8].

The virus has an icosahedral symmetry, its diameter is 70 nm non-enveloped virus. The virus has three layers of capsid arranged as an outer capsid, an inner capsid, and a core. Glycoprotein (VP7) and (protease-sensitive protein (VP4) in the outer capsid stimulate the production of neutralizing antibodies. The genotypes of rotavirus is classified according the difference in the genetic regions that encoding VP7 and VP4 to the G and P genotypes [9]. There are identified 27 G genotypes and 37 P genotypes [10]. Globally, G1, 2, 3, 4, and 9 combined with P [4] or P [8] form the most common genotypes in humans [11].

Previous studies from Egypt from different geographical locations identified rotavirus infection to be associated with genotypes of G1, G2, and G4 [12–16].

The World Health Organization (WHO) recommends the universal immunization of two approved rotavirus vaccines, RotaTeq and Rotarix, due to the significant public health burden of RV diarrheal illness, especially in low-income countries. The World Health Organization (WHO) made this recommendation in 2013. A strain called RotaTeq is a mix of G1-G4 and P [8], while Rotarix is from the P1A [8] G1 genotype [17, 18].

To diagnose rotavirus in the laboratory, antigen detection methods like enzyme-linked immunosorbent assay (ELISA) and antibody ELISA techniques are used to find the virus. In addition, there are PCR methods that exhibit excellent sensitivity and provide speedy findings. The reverse transcriptase polymerase chain reaction (RT-PCR) is a technique that utilizes generic or type-specific primers. Researchers frequently use the VP4, VP6, and VP7 areas [8, 19].

Research from Egypt was conducted to identify G genotypes of rotavirus associated with acute gastroenteritis in children; however, there are not enough studies about the P genotypes of rotavirus in Egypt. Moreover, there is an increase in vaccination for rotavirus in private health clinics that may modify the circulating genotypes of rotavirus in the community.

Therefore, the present study aimed to detect both the G and P genotypes of rotavirus in children ≤ 5 years old in one center in Egypt as a cross-sectional study, to correlate the genotypes with various demographic and clinical data in infected children and to evaluate the common mixed genotypes G and P in infected children.

## Material and method

The cross-sectional study included children with acute gastroenteritis ≤ 5 years old from January 2023 till March 2024 recruited from Mansoura University Children’s Hospital, Egypt based upon laboratory diagnosis by exclusion of bacterial and protozoa pathogens. The inclusion criteria were children with acute gastroenteritis with stool frequency > 3 times per day in the last 72 h. Children with diarrhea due to therapy or associated with other types of infections or disorders such as upper respiratory tract infections or liver disorders were excluded from the study. Moreover, children with bacterial or protozoa infestation diagnosed by laboratory methods were excluded from the study. The study was approved by Mansoura Faculty of Medicine ethical committee (R. 24.06.2665.) and approval was obtained from the parents of each participant. Each child was subjected to full clinical history taking and clinical examination including the clinical dehydration scale (CDS) for the assessment of dehydration according to a combination of the scores of general appearances, eyes, mucous membranes, and tears [20].

### Sample size

The sample size depends upon the time nonprobability sampling method the sample size depends on the time of study in which enrolled children with acute gastroenteritis with the inclusion criteria were recruited.

Clinical data was reported regarding age, sex, the associated symptoms such as fever, vomiting abdominal pain, and the season in which the patient had the manifestation of the diarrhea.

## Sample

A stool sample was obtained from each child in a clean container and transported to the laboratory for rotavirus antigen detection by Enzyme-linked Immunosorbent Assay (ELISA- RIDASCREEN). The stool samples were kept frozen at -80ºC till molecular analysis for rotavirus.

### ELISA- RIDASCREEN for rotavirus

The ready-to-use commercial kit employs monoclonal antibodies in a sandwich-type method. The monoclonal antibody is specific to the sixth viral antigen and is coating the well in the microwell plate. The prepared suspension of the stool sample and the control were added to the wells with biotinylated monoclonal anti-rotavirus antibodies and incubated at room temperature. Then wash and add streptavidin poly-peroxidase conjugate with second incubation at room temperature. In the presence of rotaviruses in the stool sample, a sandwich complex will form of immobilized antibodies, the rotavirus antigens, and the antibodies conjugated with the biotin-streptavidin-peroxidase complex. After the second wash to remove the unattached streptavidin poly-peroxidase conjugate the substrate will be added that changes the color to blue if the test is in the presence of rotavirus antigen. The addition of a stop reagent changes the color from blue to yellow. The extinction is proportional to the concentration of rotaviruses found in the specimen.

### Extraction of RNA

Stool samples were stored at − 80 °C then stool suspension was done for the preparation of 20% stool suspensions in phosphate-buffered saline for molecular assays. Rotavirus dsRNA was extracted from freshly prepared stool suspensions using the viral RNA Mini-Kit (Qiagen, Hilden, Germany) as the instruction of the manufacturer.

The extracted RNA was resuspended in 15 mL of RNase-free water. The VP7 gene was reverse transcribed and amplified using plus sense primer sBeg9 nucleotides 1–21, 5’-GGCTTTAAAAGAGAGAATTTC-3’) and minus-sense primer End (nucleotides 1062–1036, 5 -GGTCACATCATACAATTCTAATCTAAG-3’), followed by G genotyping using a cocktail of primers specific to the 7 human Rotavirus genotypes (G1–G4, G8, G9 and G12). The primers are listed in Table 1 [21].

### G genotyping nested -PCR

The prepared mixtures of the amplification mixture, cDNA and the primers in concentration mentioned previously in reference 21 were in a preheated (94ºC) Perkin Elmer thermal cycler for 10–20 PCR cycles under the following conditions: 94ºC, 10 cycles for 0.5 min 42ºC, 0.5 min 72ºC, 1 min, 1 cycle 72ºC, 7 min 1 cycle soak at 4ºC.

In the second amplification cycle aliquot 48 µl of the PCR Taq mixture into a new tube for each sample and add over it 2 µl of the centrifuged product of the first cycle.

Transfer the tubes to the thermal cycler, and run for 30 PCR cycles with following conditions Ten cycles of 0.5 min at 94 C, 0.5 min at 42 C, one minute at 72 C then one cycle 72ºC for 7 min and finally at 4ºC.

Dilute 10 µl of each PCR product in 6X gel loading buffer and electrophorese in a 1.8% agarose gel to detect products. Use 5 µl of the 123-bp DNA ladder + 5 µl of water as a marker to determine the size of the PCR products [21].

### P genotyping

The VP4 gene was amplified by RT-PCR using gene-specific primers, and P-genotypes were determined using primers specific for P [4], P [6], P [8], P [9], and P [10] as documented in the WHO manual of Rotavirus detection and characterization methods [21]. Place the amplification mixture in the preheated (94ºC) Perkin Elmer thermal cycler then the following PCR conditions: 20 cycles 94ºC, 0.5 min, 42ºC, 0.5 min 72ºC, 1 min, then 1 cycle 72ºC, 7 min 1 cycle Soak at 4ºC.

In the second amplification cycle aliquot 48 µl of the PCR Taq mixture into a new tube for each sample and add 2 µl of the centrifuged product of the first cycle.

Run 30 cycles of PCR with the following conditions Twenty cycles, 94ºC for 0.5, 42ºC for 0.5, 72ºC for 0.75, one cycle 72ºC for 7 min, then 4ºC for 1 min (21).

**Table 1:** Sequences used in the amplification of genotypes G and P with the amplified product sizes base pair (bp)

	Sequences	bp
First amplification		
con1	5^/^-TAGCTCCTTAATGTATGG-^/^3	
con2	5^/^-GTATAAAATACTTGCCAC-^/^3	
Second-amplification genotyping primers		
T1	5^/^-TCTTGTCAAAGCAAATAATG-^/^3	749 bp
T2	5^/^-GTTAGAAATGATTCTCCACT-^/^3	652 bp
T3	5^/^-GTCCAGTTGCAGTGTAGC-^/^3	bp 812
T4	5^/^-GGGTCGATGGAAAATTCT-^/^3	583 bp
T9	5^/^-TATAAAGTCCATTGCAC-^/^3	306 bp
First amplification consensus		
con3	5^/^-TGGCTTCGCTCATTTATAGAC-^/^3	
con2	5^/^-ATTTCGGACCATTTATAACC-^/^3	
1T-8	5^/^-TCTACTTGGATAACGTGC-^/^3	224 bp
2T-4	5^/^-CTATTGTTAGAGGTTAGAGTC-^/^3	362 bp
3T-6	5^/^-TGTTGATTAGTTGGATTCAA-^/^3	146 bp
4T-9	5^/^-TGAGACATGCAATTGGAC-^/^3	270 bp
5T-10	5^/^-ATCATAGTTAGTAGTCGG-^/^3	462
ND2	5^/^-AGCGAACTCACCAATCTG-^/^3	

### Statistical analysis

The data was analyzed by the SPPS24 program. The numerical data was expressed as minimum, maximum, and median for nonparametric data. The qualitative data was expressed as numbers and percentages. The comparison of qualitative data was performed by Chi-square test and P was considered significant if < 0.05.

## Result

The study included 189 children with acute gastroenteritis. There were 108 males (57.1%) and 81 females (42.3%) with a minimum age of 0.3 years, a maximum age of 5 years, and a median age of 1.00 years. The children were mainly from rural regions (57.7%) during summer (50.2%). The common presenting signs were fever (57.7%) and abdominal pain (37%). The dehydration was uncommon sign with only 14.9% with severe dehydration and some dehydration was reported in 36.0%, Table 2.

**Table 2:** Demographic and clinical data of the studied patients

Data	
**Age (years)**	
Minimum	0.3
Maximum	5.00
Median	1.00
**Sex**	
Male (No.-%)	108 57.1%
Female (No.-%)	81 42.9%
**Residence**	
Rural (No.-%)	109 57.7%
Urban(No.-%)	80 42.3%
**Season**	
Summer (No.-%)	95 50.3%
Spring (No.-%))	52 27.5%
Autumn (No.-%))	27 14.3%
Winter (No.-%)	15 7.9%
**Abdominal pain (No.-%)**	70 37%
**Fever (No.-%)**	109 57.7%
**Dehydration**	
No dehydration (No.-%)	94 49.7%
Moderate (No.-%)	68 36.0%
Severe (No.-%)	27 14.3%

A nested PCR study for rotavirus genotypes revealed that G1 was the most common genotype (24.7%) followed by G2 (21.1%), G3 (20%), G9 (20%), and G4 (14.1%). The genotyping of the P genotype revealed that P9 was the commonest genotype (24.7%), followed by P4 (21.2%), P10 (20%), P8 (17.6%) and P6 (16.5%), Table 3.

**Table 3:** Genotypes G and P of rotavirus by nested PCR

Genotypes of Rotavirus	Rotavirus Positive by antigen (*n* = 85)	
	No.	%
**Genotypes G**		
G1	21	24.7%
G2	18	21.1%
G3	17	20%
G4	12	14.1%
G9	17	20%
**Genotype P**		
P4	18	21.2%
P6	14	16.5%
P8	15	17.6%
P9	21	24.7%
P10	17	20%

*P* = 0.001

Chi-square test

The positive rotavirus antigen was more frequently detected in females (55.3%) than males (44.7%, Odd ratio 0.2, 95% CI 0.22–0.71, *P* = 0.001). There was a significant association between the summer season and positive rotavirus antigen (*P* = 0.001) and rural residence of the patients (Odd ratio 6,9 95%CI 3,5-13.5, *P* = 0.001). The significant associated clinical sign with positive rotavirus antigen was fever (Odd ratio 3,3, 95%CI 1,8-6.05, *P* = 0.001). The genotypes G and P were significantly associated with positive rotavirus antigen as all cases positive by antigen had been detected by nested PCR with the commonest genotypes G4 (24.7%, *P* = 0.001) and genotype P9 (24.7%, *P* = 0.001), Table 4.

**Table 4:** Comparison between demographic data, clinical data, and genotypic in children with rotavirus antigen positive versus children with negative rotavirus

Data	Rotavirus Positive by antigen (*n*=85)		Rotavirus Negative by antige (*n*=104)		Odd Ratio	95% Confidence Interval	*P*
	No.	%	No.	%			
**Sex**					**0.2**	0.22-0.71	
Male	38	44.7%	**70**	**67.3%**			*P*=0.001
Female	47	55.3%	**34**	**32.7%**			
**Season**							
Summer	77	90.6**%**	18	17.3%			*P*=0.001
Spring	4	4.7%	48	46.1%	-	-	
Winter	4	4.7%	11	10.6%			
Autumn	0	0%	27	25.96%			
**Abdominal pain**	30	35.3%	40	38.5%	0.87	0.48-1.59	*P*=0.38
**Fever**	62	72.9%	47	45.2%	3.3	1.8-6.05	*P*=0.001
**Dehydration**							*P*=0.8
No	42	49.4%	52	50%	-	-	
Some dehydration	32	37.6%	36	34.6%			
Severe dehydration	11	12.9%	16	15.4%			
**Residence**							*P*=0.001
Rural	69	81.2%	40	38.5%	6.9	3.5-13.5	
Urban	16	18.8%	64	61.5%			
**Genotypes**							
G					-	-	*P*=0.001
G1	**21**	**24.7%**	**0**	**0%**			
G2	**18**	**21.1%**	**0**	**0%**			
G3	**17**	**20%**	**0**	**0%**			
G4	**12**	**14.1%**	**0**	**0%**			
G9	**17**	**20%**	**0**	**0%**			
**Genotype P**							
P4	**18**	**21.2%**	**0**	**0%**	-	-	*P*=0.001
P6	**14**	**16.5%**	**0**	**0%**			
P8	**15**	**17.6%**	**0**	**0%**			
P9	**21**	**24.7%**	**0**	**0%**			
P10	**17**	**20%**	**0**	**0%**			

Chi-square test

The commonest combined genotypes of G and P were G1P4 (85.7%), G3P8(88.2%), followed by G2P6 (77.8%) and G9P9(76.5%) and G4P9 (66.7%) followed by G4P10 (33.3%), G9P10(23.5%), G2P10(22.2%), G1P10 (14.3%),G3P10(11.8%). The distribution was significant (*P* = 0.001), Table 5.

**Table 5:** Association between different genotypes of rotavirus

	G1 (*n* = 21)		G2 (*n* =18)		G3 (*n* = 17)		G4 (*n* = 21)		G9 (*n* = 17)	
	No.	%	No.	%	No.	%	No.	%	No.	%
P4	18	85.7%	0	0%	0	0%	0	0%	0	0%
P6	0	0%	14	77.8%	0	0%	0	0%	0	0%
P8	0	0%	0	0%	15	88.2%	0	0%	0	0%
P9	0	0%	0	0%	0	0%	8	66.7%	13	76.5%
P10	3	4.3%	4	22.2%	2	11.8%	4	33.3%	4	23.5%

*P* = 0.001

## Discussion

Diarrhea is a major worldwide cause of illness and death in children under the age of five, with rotaviruses being the primary culprit [22]. The objective of this study was to identify the genotypes of rotavirus in children ≤ 5 who are experiencing diarrhea at a specific center in Egypt. A nested PCR study for rotavirus genotypes revealed that G1 was the most common genotype (24.7%) followed by G2 (21.1%), G3 (20%), G9 (20%), and G4 (14.1%). The genotyping of the P genotype revealed that P9 was the commonest genotype (24.7%), followed by P4 (21.2%), P10 (20%), P8 (17.6%) and P6 (16.5%).

The study findings indicate that a majority (57.7%) of the children included in the study are from rural areas. This suggests that there may be differences in sanitation, availability of clean water, healthcare facilities, and dietary habits when compared to urban areas [23]. Several variables may contribute to the elevated prevalence of acute diarrhea in specific regions. According to the data, summer accounted for most cases, with a proportion of 50.2%. There are other potential causes for this occurrence, such as increased consumption of perishable foods, changes in the origin of drinking water, or variations in the prevalence of disease-causing microorganisms associated with diarrhea during different times of the year [24].

Gaining insight into the demographic characteristics of acute diarrhea could assist healthcare providers and governments in customizing treatments and efficiently distributing resources. For example, directing efforts toward enhancing sanitation infrastructure in rural regions or executing specific health education initiatives during the periods of increased vulnerability could effectively decrease the prevalence of diarrhea in children. Although the study offers intriguing insights, it is crucial to take into account potential limitations, including the sample size, geographic breadth, and the methodologies employed for data collection and analysis.

Fever was the primary clinical indicator that strongly correlated with a positive rotavirus antigen (*P* = 0.001). This suggests that there is rotavirus viremia in those patients [25].

The novelty of the present study was the determination of rotavirus genotypes regarding G and P in one Egyptian center where there is limited practice of vaccination against rotavirus. The genotype findings can be used to be incorporated into vaccination to prevent rotavirus infection.

The results of the nested PCR analysis for rotavirus genotypes showed that G1 was the predominant genotype, accounting for 24.7% of the samples. G2 followed at 21.1%, G3 at 20%, G9 at 20%, and G4 at 14.1%. The results of the nested PCR analysis on rotavirus genotypes provide valuable information regarding the frequency and distribution of different genotypes within the studied population. Global epidemiological evidence consistently acknowledges G1 as the dominant genotype, recognizing it as one of the main rotavirus strains worldwide. The high frequency of this agent emphasizes its significance as a main contributor to rotavirus infections [26].

Although we commonly acknowledge rotavirus genotypes G2, G3, and G9, their prevalence may vary across different areas and periods due to the introduction of regular immunization. A previous meta-analysis study identified serotype G9 as the predominant genotype of RV, representing 85.48% of all infections. According to Li (2024) (27), G2 had a percentage of 7.70%, G8 had a percentage of 5.74%, G1 had a percentage of 4.86%, and G3 had a percentage of 3.21%. Previous meta-analyses conducted before the introduction of vaccination showed that the most common G genotypes were G1 (48%), followed by G2 (19%) and G3 (12%) (28). The present study’s findings revealed that G1, G2, and G3 are the most prevalent strains in our region. This prevalence remains unchanged due to the limited immunization practices in private clinics.

The genotyping analysis of the P genotype showed that P9 was the most prevalent genotype, accounting for 24.7% of the samples. P4 followed at 21.2%, P10 at 20%, P8 at 17.6%, and P6 at 16.5%.

A previous meta-analysis, on the other hand, found that the most common P genotype was P [8], which accounted for 64.02% of rotavirus cases in places where people get rotavirus shots regularly [27, 28].

P9 is the most prevalent P genotype in the research population, much like G1. P9 frequently co-occurs with G1 rotavirus strains, suggesting a shared genotype combination that could be accountable for a substantial number of rotavirus infections. A study conducted in Poland found that the P9 genotype of rotavirus was the most common [29]. All strains of feline, canine, and feline-like human rotavirus (HRS) exhibited significant genetic variation. This variation is mostly due to the occurrence of numerous transmissions between different species, combined with reassortment events, which have a significant impact [30]. These findings emphasize the importance of closely and simultaneously monitoring RVs in both animals and humans, as animal RVs have demonstrated the ability to induce severe illness in humans [31–33]. The study determined that P4 was another common P genotype. Like G1, P4 is frequently associated with rotavirus infections and has a significant global impact on disease. Djojosugito, Fauzia, et al. (2017) [34] conducted a study in Indonesia and found that P4, P8, and P6 were the most prevalent genotypes. The research population likewise exhibits these P genotypes, although their frequencies are slightly lower in comparison to P9 and P4. Their existence emphasizes the genetic variety of rotavirus strains that are now spreading in the community, as well as the importance of monitoring various genotypes to create effective vaccines and therapies that target specific strains.

The most often observed combination genotypes of G and P were G1P4 (85.7%), G3P8 (88.2%), G2P6 (77.8%), G9P9 (76.5%), G4P9 (66.7%), G4P10 (33.3%), G9P10 (23.5%), G2P10 (22.2%), G1P10 (14.3%), and G3P10 (11.8%). The distribution exhibited statistical significance with a p-value of 0.001.

Consistent with our findings, prior research indicated that G1P4 was a prevalent rotavirus genotype [35, 36]. In contrast, Antonie et al. (2023) [37] found that G1P [8] was the predominant genotype in the African Region and the second most common in the European Region. The prevalence of G1P [8] genotypes was significantly lower in countries that had implemented the rotavirus vaccine compared to countries that had not yet introduced the vaccine in both regions. The contrast was especially pronounced in the European Region, with a 33% against 8% disparity. Since 2006, the majority of nations in the Americas have been using the rotavirus vaccine. As a result, the G1P [8] genotype of the rotavirus has become extremely rare, with a prevalence of less than 1%. The Eastern Mediterranean Region, which encompasses two nations that have already incorporated the vaccine into their immunization schedules, showed a rather small percentage (6%) of this genotype. This finding aligns with earlier research that has shown a shift from the most common genotype (G1P [8]) to other genotypes after the introduction of the vaccination. Additionally, there is now a wider variety of genotypes present. Findings from the African and European regions back up earlier proof that the Rotarix^®^ and Rotateq^®^ vaccines work against the G1P genotype [38]. The rotavirus vaccine is now not part of the national immunization program in Egypt; however, it is available through private health providers.

These findings suggest that the predominance of RV genotypes can occur in different combinations. Rotavirus genotypes G1P4, G1P6, G4P6, G9P4, G12P6, and G6P4 in animals may exhibit periodic bouts of recurring appearance or disappearance. While the predominant G/P combinations reported in various geographical regions may seem similar, the proportions of these combinations vary among geographic regions and change over time. A recent study by Bukhari et al. (2022) [39] showed that analyzing the diversity of rotavirus strains and effectively managing patients with diarrhea can help identify a range of newly developing strains.

Since nested PCR also identified all patients who tested positive for the antigen, genotypes G and P showed a strong correlation with the presence of rotavirus antigen. Rotavirus antigen assays exhibit a high level of sensitivity in accurately diagnosing rotavirus infection in children with acute gastroenteritis (AGE). Furthermore, these tests can be useful for identifying and clinically managing rotavirus infection in children and preventing the onset of the illness [40].

## Conclusion

The present study highlights the common rotavirus genotypes at one center in Egypt, G1, G2, and G3 were the commonest G genotypes. Regarding genotype P the commonest genotypes were P9, P4, and P10. The commonest combined genotypes were G1P4, G3P8, G2P6. There was no effect of the practice of rotavirus vaccination at limited rates at private health sections as the rotavirus is still a major pathogen of acute gastroenteritis in children. There is a need for the inclusion of rotavirus vaccination in the national program of children vaccination in Egypt.

## Data Availability

The datasets generated and analyzed during the current study are available in the Fighshare repository at 10.6084/m9.figshare.26114743.v1.
